# Dynamics of Functional Genes and Bacterial Community during Bioremediation of Diesel-Contaminated Soil Amended with Compost

**DOI:** 10.4014/jmb.2210.10038

**Published:** 2023-02-10

**Authors:** Hyoju Yang, Jiho Lee, Kyung-Suk Cho

**Affiliations:** Department of Environmental Science and Engineering, Ewha Womans University, Seoul 03760, Republic of Korea

**Keywords:** Bioremediation, compost, diesel-contaminated soil, functional gene, bacterial community, greenhouse gas

## Abstract

Compost is widely used as an organic additive to improve the bioremediation of diesel-contaminated soil. In this study, the effects of compost amendment on the remediation performance, functional genes, and bacterial community are evaluated during the bioremediation of diesel-contaminated soils with various ratios of compost (0–20%, w/w). The study reveals that the diesel removal efficiency, soil enzyme (dehydrogenase and urease) activity, soil CH_4_ oxidation potential, and soil N_2_O reduction potential have a positive correlation with the compost amendment (*p* < 0.05). The ratios of denitrifying genes (*nosZI*, *cnorB* and *qnorB*) to 16S rRNA genes each show a positive correlation with compost amendment, whereas the ratio of the CH_4_-oxidizing gene (*pmoA*) to the 16S rRNA genes shows a negative correlation. Interestingly, the genera *Acidibacter*, *Blastochloris*, *Erythrobacter*, *Hyphomicrobium*, *Marinobacter*, *Parvibaculum*, *Pseudoxanthomonas*, and *Terrimonas* are strongly associated with diesel degradation, and have a strong positive correlation with soil CH_4_ oxidation potential. Meanwhile, the genera *Atopostipes*, *Bacillus*, *Halomonas*, *Oblitimonas*, *Pusillimonas*, *Truepera*, and *Wenahouziangella* are found to be strongly associated with soil N_2_O reduction potential. These results provide useful data for developing technologies that improve diesel removal efficiency while minimizing greenhouse gas emissions in the bioremediation process of diesel-contaminated soil.

## Introduction

Total petroleum hydrocarbons (TPHs) are major components of petrochemicals and are used in various industrial fields [1]. Upon leakage during storage, transportation, and use, TPHs become notable sources of soil and groundwater contamination [1-3]. Hence, bioremediation is attracting attention as an eco-friendly and economical technology for the remediation of TPH-contaminated soil [4-6]. This process has the advantage that secondary pollution due to by-products hardly occurs because the TPHs are decomposed into carbon dioxide and water by the microorganisms [7-12]. Nevertheless, the metabolic activities of the microorganisms in the TPH-contaminated soil tend to be low as the TPHs are mainly composed of recalcitrant carbon- and hydrogen-containing compounds, such as long-chain aliphatic and aromatic hydrocarbons [13]. Bioremediation performance can therefore be improved by using chemical or organic additives to supply nitrogen, phosphorus, and growth factors for the soil microorganisms [4, 14].

Compost is widely used as an organic additive to improve the bioremediation performance of TPH-contaminated soil [15-18]. Compost can provide not only nutrients such as nitrogen, phosphorus, and other minerals, but also microbial sources [15-18]. Compost amendment changes the soil microbial activity and microbial community related to the metabolic pathways of carbon and nitrogen compounds. Therefore, compost amendment can influence CH_4_ and N_2_O emissions as well as the removal of TPHs during bioremediation. In one study, the bioremediation efficiency of crude oil-contaminated soil was improved by more than 29 times by the addition of compost [19]. Moreover, when compost was added to diesel-contaminated soil, the abundance of the *alkB* gene, a functional gene contributing to alkane decomposition, increased [18]. In another study, the addition of compost to diesel-contaminated soil not only enhanced the dominance of the methane-oxidizing microorganism community, but also increased the abundance of the *pmoA* gene, a functional gene contributing to methane oxidation, relative to that of soil treated with chemical nutrients [18]. Lee *et al*. [20] reported that when compost was added to diesel-contaminated soil planted with maize, the abundances of the *cnorB* and *nosZ* genes, functional genes involved in NO and N_2_O reduction, increased compared to the control in the absence of compost.

In spite of the above-mentioned studies, there remains a lack of information on the effects of compost amendment upon the dynamics of the microbial community, and the functional genes associated with the degradation of TPHs and the emission of CH_4_ and N_2_O, during the bioremediation of TPH-contaminated soil. Therefore, the present study evaluates the TPH removal, soil enzyme activity, CH_4_-oxidizing, and N_2_O-reducing potentials of TPH-contaminated soils amended with various ratios (0–20%, w/w) of compost. During the bioremediation period, the functional genes associated with TPH degradation (namely, *alkB* and *CYP153*), CH_4_ production (*mcrA*), CH_4_ oxidation (*pmoA*), N_2_O production (*cnorB*, *qnorB*), and N_2_O reduction (*nosZI*) are determined via the quantitative polymerase chain reaction (qPCR). Moreover, the dynamics of the soil bacterial community are characterized via a high-throughput sequencing method (Miseq, Illumina Inc., USA). Finally, based on correlation and network analyses, the key parameters that are highly affected by compost amendment in the bioremediation of TPH-contaminated soil are clarified.

## Materials and Methods

### Materials and Soil Preparation

Coarse sand (2 mm in diameter, Kimhae Masato, Korea) and perlite (Kyungdong One Co. Ltd., Korea) were mixed in a 4:1 ratio (v/v) to generate barren soil. Various amounts of compost (0, 5, 10, and 20 wt.%) were added to the barren soil to produce mixtures designated as S-C0 (control), S-C5, S-C10, and S-C20, respectively. The compost was prepared by a commercial vendor (Seokgang Green Fertilizer Inc., Korea) via the fermentation of swine manure (40%), sawdust (29%), cow manure (10%), and bacterial inoculum (1%). After purchase, the soil samples were artificially contaminated with diesel (10,000 mg diesel/kg soil). The physicochemical properties of the barren soil and compost were analyzed by the National Instrumentation Center for Environmental Management (NICEM), Republic of Korea (Table 1). The textures of the barren soil and compost were classified as sandy loam and sand, respectively. The moisture and organic contents of the barren soil were 2.9 and 0.2%, respectively. The total nitrogen, ammonium nitrogen, and nitrate nitrogen concentrations of the compost were 26,000, 465.3, and 34.5 mg N/kg soil, respectively. The total phosphate content of the compost was significantly high (11,443.8 mg P/kg soil). The moisture and organic contents of the compost were 55.3 and 36.7%, respectively.

### Pot Experiment and Soil Sampling

The pot-scale experiment was conducted for 103 days (from June 3^rd^ to September 14^th^, 2021) in the rooftop garden of the new engineering building at Ewha Womans University (37° 56¢ 65¢¢ N, 126° 94¢ 85¢¢ E). The processes of soil sample preparation and pot setting are shown photographically in Fig. S1 of the Supplementary Material. After setting a layer of coarse sand up to 1 cm from the bottom of each pot (18 cm in diameter × 15 cm in height), each soil mixture amended with 0–20 wt.% compost was added into its own pot so that the soil layer was 14 cm in height (Figs. S1B and S1D). The lower parts of the pots were then buried in the garden to avoid the intense solar radiation (Fig. S1C). Each pot experiment was conducted in duplicate. The pot soil was watered 3 times a week to maintain an average moisture content of 14.58%, and the soil mixture in the pot was manually mixed every other day using a trowel.

Soil sampling was performed on days 0, 12, 33, 51, 76, and 103 according to the United States Environmental Protection Agency (US EPA) method, as modified by Hu *et al*. [21]. The soil in the pot was mixed well before sampling, and 200 g of soil was randomly collected in a polyethylene bag. After further thorough mixing, 50 g of the collected soil was placed in each of two conical tubes, one of which was then stored at –23°C for subsequent DNA extraction, and the other stored at –80°C for measuring the residual TPH concentrations. The remaining soil sample in the polyethylene bag was stored at 4°C for use in analyzing the moisture content, organic content, pH, water holding capacity (WHC), and microbial enzyme activity. The analyses of moisture content, organic content, and pH were performed within 4 h of soil sampling, and the microbial enzyme activity was measured within a week.

### Analysis of Soil Properties and Residual Diesel Concentrations

The moisture contents of 3-g soil samples were measured by drying each sample at 110°C for 4 h, and the organic contents of 3-g samples were measured by drying at 550°C for 2 h. The moisture and organic contents were then calculated from the weight differences before and after heating [18]. To measure the pH of the soil, a 5-g sample was mixed with distilled water (25 ml), and then kept at room temperature for 1 h [22]. The pH of the soil suspension was then measured using a pH meter (Orion 420A, Thermo Scientific Inc., Japan). The WHC of the soil was analyzed using the method described by Vengadaramana and Jashothan [23]. All experiments were performed in triplicate.

After freeze-drying the soil samples according to the method of Lee *et al*. [20], their residual diesel concentrations were measured by first thoroughly mixing the 3-g samples with 1:1 (v/v) hexane:acetone solution (5 ml) in a 15-ml test tube and shaking for 30 min at 30°C to extract the residual diesel. Then, an organic-phase aliquot (1.5 ml) was transferred to a 2-ml vial, and a 1-μl sample of this aliquot was analyzed using a gas chromatograph (6890N, Agilent Technologies, CA, USA) [20, 24]. The residual diesel analysis was performed in 5 replicates. The removal efficiency of diesel was calculated according to Eq. (1):

$$\text{RE (\%) = (}C_{0}-C_{i})/C_{0}\times 100,$$ (1)

where C_0_ is the initial concentration, and C_i_ is the concentration on the *i*^th^ day.

As a result of diesel extraction using the same method for the soil without the addition of diesel (negative control), the diesel concentration was below the detection limit.

### Soil Enzyme Activity

To evaluate the effect of compost amendment on the soil enzyme activity during the bioremediation of diesel-contaminated soil, the dehydrogenase activity (DHA) and urease activity (UA) were measured. For measuring the DHA, 2 g of the soil was mixed with 2 ml of Tris-HCL buffer (pH 7.6) and 1 ml of 1% (w/v) triphenyl tetrazolium chloride (TTC) solution in a test tube, and incubated in the dark at 37°C for 24 h. Then, 96% ethanol (10 ml) was added, and the mixture was shaken and transferred to a 15-ml centrifuge tube for centrifugation at 3,000 *g* for 5 min. The concentration of the produced triphenyl formazan (TPF) in the supernatant was then measured using a spectrophotometer (Libra S22, Biochrom, UK) at 485 nm. The DHA was then determined as the amount of TPF (in mg) produced per 1 gram of dry soil per hour (mg TPF/g dry soil/h) [6].

For measuring the UA, the soil sample (2 g) was mixed with 0.08 M urea solution (0.5 ml) and incubated for 2 h at 37°C in a standing incubator. Then, 1 M KCl/HCl solution (10 ml) was added, and the mixture was stirred at 180 rpm and 25°C for 30 min. The mixture was then transferred to a 15-ml centrifuge tube and centrifuged at 3,000 g for 5 min. Next, the supernatant (3 ml) was mixed gently with distilled water (1.2 ml), 12% sodium phenolate solution (0.5 ml), 0.15% sodium nitroprusside solution (50 μl), and 1% NaOCl solution (0.25 ml), and allowed to stand at room temperature for 30 min. After that, a 1 ml aliquot was examined using a Libra S22 spectrophotometer (Biochrom) at 630 nm, and the UA was determined as the amount of ammonium (in μg) produced per 1 gram of dry soil per hour (μg NH_4_^+^-N/g dry soil/h) [25].

### Evaluation of Soil CH_4_ Oxidation and N_2_O Reduction Potentials

The effects of compost amendment upon the CH_4_ oxidation and N_2_O reduction potentials of diesel-contaminated soil during bioremediation were investigated using the same methods as in previous studies [24, 26, 27]. Thus, the soil sample (2 g) was inoculated into nitrate minimal salt medium (NMS, 6 ml) in a 120-ml serum bottle [24, 27]. The bottle was then sealed with a butyl rubber cap, and 50,000 ppm (v/v) of CH_4_ gas (99.99%, Dong-A Specialty Gases Co., Korea) was injected into the headspace of the bottle. While incubating the bottle at 30°C and 150 rpm, the CH_4_ concentration in the headspace was measured using a gas chromatograph (7890A, Agilent Technologies, USA) [24, 27].

To evaluate the soil N_2_O reduction potential, the soil sample (5 g) was inoculated into mineral medium (30 ml) in a 600-ml serum bottle and sealed with a butyl rubber cap [26]. After purging with N_2_ gas (99%, Dong-A Specialty Gases), the glucose and acetate solution (100 mg COD/L) was added into the bottle, followed by the injection of 1,000 ppm (v/v) of N_2_O gas (99.99%, Dong-A Specialty Gases) into the headspace. While incubating the bottle at 30°C and 150 rpm, the N_2_O concentration in the headspace was measured using a gas chromatograph (7890B, Agilent Technologies) [26].

### Functional Gene Abundance Analysis

The qPCR was performed to evaluate the change in functional gene abundances during the bioremediation of diesel-contaminated soil amended with various ratios of compost. For this procedure, DNA was extracted from the soil sample using a NucleoSpin Soil Kit (Macherey-Nagel GmbH, Germany) [27]. The 16S rRNA gene was quantitatively evaluated for total bacteria abundances using 340F and 805R primer sets [28]. The alkane monooxygenase (*alkB*) and *CYP153* gene abundances associated with diesel degradation were quantified using *alkB*-1F/*alkB*-1R [29] and *CYP153*_C4_F/*CYP153*_C4_R [30] primer sets, respectively. The abundances of the particulate methane monooxygenase (*pmoA*) gene associated with CH_4_ oxidation, and of the methane reductase subunit A (*mcrA*) gene involved in CH_4_ production, were measured using A189f/mb661r [31] and mlas/*mcrA*-rev [32] primer sets, respectively. The abundances of the nitric oxide reductase genes *qnorB*, *cnorB*, and *nosZI*, that are associated with N_2_O production and reduction, were quantified using the *qnorB*-2F/*qnorB*-7R [33], norB1-F/norB6-R [33], and *nosZ* 1F/*nosZ* 1R [34] primer sets, respectively. The solution compositions and reaction conditions for the qPCR are listed in Table S1.

### Bacterial Community Analysis

The extracted DNA was used as a PCR template to analyze the bacterial community with an Illumina Miseq sequencing platform (Macrogen Inc., Korea) by the method reported previously [20]. The PCR was performed to amplify the 16S ribosomal RNA gene containing the V4 region using the 515F/806R primer set [20]. The sequences were analyzed via the Illumina MiSeq sequencing platform purchased from Macrogen Inc. The sequence reads were analyzed using the QIIME software version 1.9 by Macrogen Inc. [35]. Sequences that were too short to contain the target base pairs were removed by using the Fast Length Adjustment of Short reads (FLASH) software version 1.2.11 [36]. Ambiguous and chimeric sequences were then removed, and the remaining sequences were classified into operational taxonomic units (OTUs) at 97% similarity using the CD-HIT-OTU program [37]. The taxonomy for each OTU was assigned based on the National Center for Biotechnology Information (NCBI) 16S microbial database. The obtained sequencing reads were deposited to the NCBI Sequence Read Archive (http://www.ncbi.nlm.nih.gov/) under accession number SRP339855. Finally, the Chao1, Shannon, and Simpson indices were calculated using the QIIME software version 1.9.

### Statistical Analysis

The *t*-tests and multiple comparisons were conducted using the R software package (www.rstudio.com), with a p-value of 0.05 indicating a significant difference. The bacterial community structures were compared by correlation analysis and principal component analysis (PCA) using the R software package and the CANOCO 4.5 software (Microcomputer Power, Ithaca, NY, USA) [38]. The heatmap for the bacterial community was visualized using the gplots tool in the R software package. The Pearson correlations between parameters were also calculated using the R software package. The extended local similarity analysis (eLSA) was performed at *p* < 0.05 to evaluate the correlation between parameters, and the results were visualized using the Cytoscape program version 3.4.0 (Institute for Systems Biology, USA).

## Results

### Physicochemical Parameters and Diesel Removal Efficiency

The variations in ambient temperature, precipitation, soil organic matter content, and soil pH with time during the pot experiments are presented in Fig. S2. The ambient temperature and precipitation information were obtained from the Korea Meteorological Administration. The average ambient temperature ranged from 13.3 to 30.2°C, and the maximum and minimum temperatures were 35.4 and 8.5°C, respectively (Fig. S2B). Among the total of 52 rainfall events, there were 3 intensive rainfalls (> 90 mm) between days 50 and 69 (Fig. S2B). The average organic contents of the soil samples increased with increasing compost addition, and were 0.64, 2.50, 3.19, and 6.07% for the S-C0, S-C5, S-C10, and S-C20 samples, respectively. These values did not change significantly during the experimental period (Fig. S2C). Meanwhile, the average pH values of the soil samples decreased with increasing amounts of added compost, being 9.01, 8.10, 7.80, and 7.76 for the S-C0, S-C5, S-C10, and S-C20 samples, respectively, and did not significantly vary during the experiment (Fig. S2D).

**Fig. 1:**
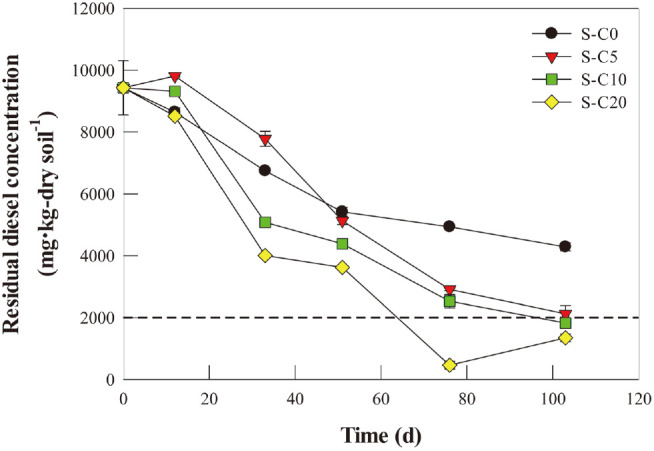
Changes in residual diesel concentration in the variously treated soil samples with time. The dotted line indicates the soil pollution standard of the Korean Ministry of Environment (*i.e.*, 2,000 mg-diesel·kg-soil^–1^).

The changes in the residual diesel concentrations of the various samples with time are presented in Fig. 1. Thus, the initial TPH concentration was 9,432 mg TPH/kg dry soil, and did not change significantly until day 12 in all samples. Thereafter, the residual diesel concentration decreased significantly, and the diesel removal rate was proportional to the amount of compost added. On day 103, the diesel removal efficiencies of the S-C0, S-C5, S-C10, and S-C20 samples were 54.6, 77.5, 80.7, and 85.7%, respectively. Notably, the residual diesel concentration of the S-C20 sample on day 76 was below the 2,000 mg TPH/kg soil pollution risk criterion for oil-contaminated soil in Korea.

### Soil Enzyme Activity

**Fig. 2:**
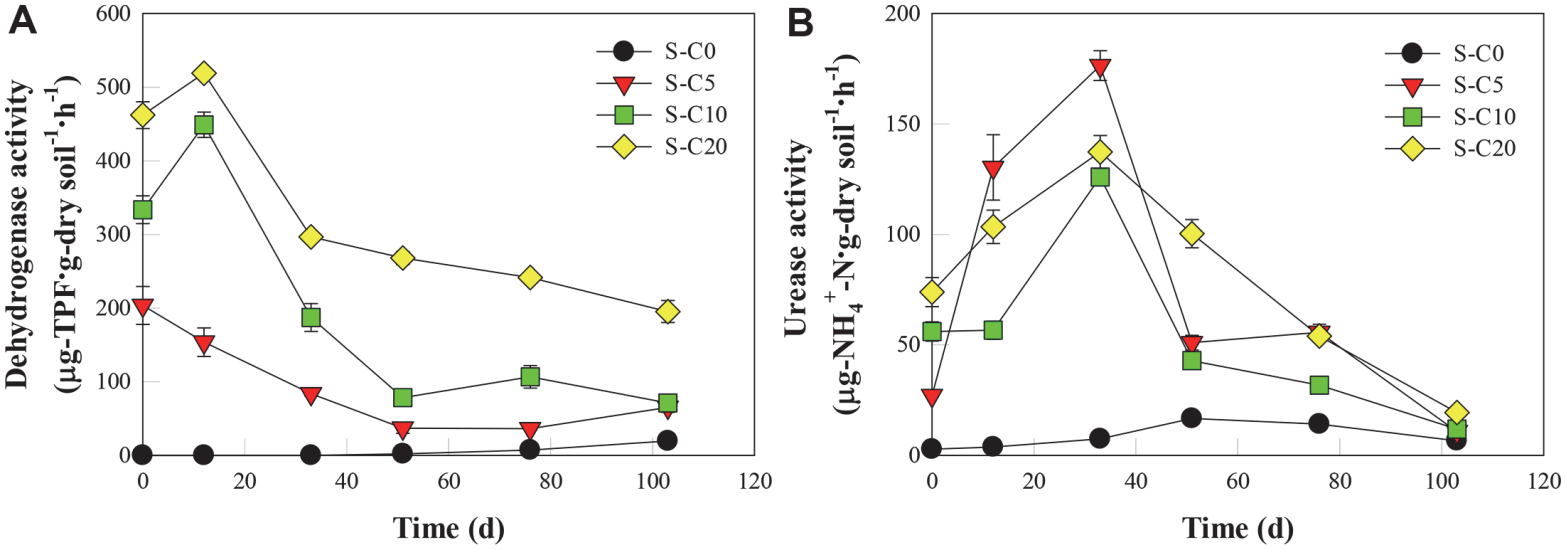
Changes in the activity of (A) dehydrogenase and (B) urease during the bioremediation of diesel-contaminated soil.

Dehydrogenase is known to be involved in the initial decomposition of soil organics, catalyzing the removal of hydrogen from organic molecules; hence, the dehydrogenase activity (DHA) is used as an index for evaluating the degradation activity of soil organics [39]. The results in Fig. 2A indicate that the DHA of the S-C0 did not significantly change during the initial 33 days, but increased slightly to 19.5 μg TPF/g dry soil/h on day 103. In the soils amended with compost, the initial DHA increased with increasing amounts of added compost, being 203.8, 333.6, and 462.2 μg TPF/g dry soil/h in the S-C5, S-C10, and S-C20, respectively (Fig. 2A). During the experimental period, the DHAs of the amended soils decreased gradually as the residual diesel concentration decreased (Figs. 1 and 2A).

Urease promotes the mineralization of organic nitrogen to hydrogen-bound nitrogen, thereby providing the soil microorganisms with ammonia as an available nitrogen source [40]. Although the urease activity (UA) cannot explain all of the biological mechanisms, it can be used as a good indicator of TPH metabolism in the soil under various soil conditions [41-43]. As with the DHA, the UA of the S-C0 sample did not change significantly during the early stages of the experiment, but increased slightly during the mid-late period (Fig. 2B). In the amended soils, the initial UA increased with increasing amounts of added compost, and further increased with time until the 33rd day, decreasing gradually thereafter (Fig. 2B).

### CH_4_ Oxidation and N_2_O Reduction Potentials

The results in Fig. S3 and Table 2 indicate that there was no significant difference in the initial CH_4_ oxidation potential of the various soil samples, which ranged from 1.40 to 1.95 μmol/g dry soil/h. During the experiment, however, the CH_4_ oxidation potential of the S-C0 increased significantly until around day 33, and remained relatively constant thereafter. Except on day 12, the CH_4_ oxidation potential of the soils amended with compost (7.00–9.83 μmol/g dry soil/h) were higher than that of the non-compost-amended soil (5.39–5.80 μmol/g dry soil/h), and continued to increase significantly with time up until at least day 51. Further, the CH_4_ oxidation potential of the S-C20 sample (8.49–9.83 μmol/g dry soil/h) was slightly higher than those of the S-C5 and S-C10 samples (7.00–8.21 μmol/g dry soil/h).

The results in Fig. S4 and Table 3 indicate that the initial N_2_O reduction potential of the non-compost amended soil (S-C0) was insignificant (< 56.82 nmol/g dry soil/h), while those of the S-C5, S-C10, and S-C20 were 868.03, 1,399.57 and 1,757.76 nmol/g dry soil/h, respectively. Moreover, while the N_2_O reduction potential gradually decreased with time during bioremediation, a relatively high activity was maintained when the amount of compost added was large. In the S-C20 sample, the N_2_O reduction potential decreased from 838.14 nmol/g dry soil/h on day 12, to 224.08 nmol/g dry soil/h on day 103. In the S-C5 sample, it decreased from 328.57 nmol/g dry soil/h on day 12, to 140.37 nmol/g dry soil/h on day 103.

### Functional Gene Dynamics

**Fig. 3:**
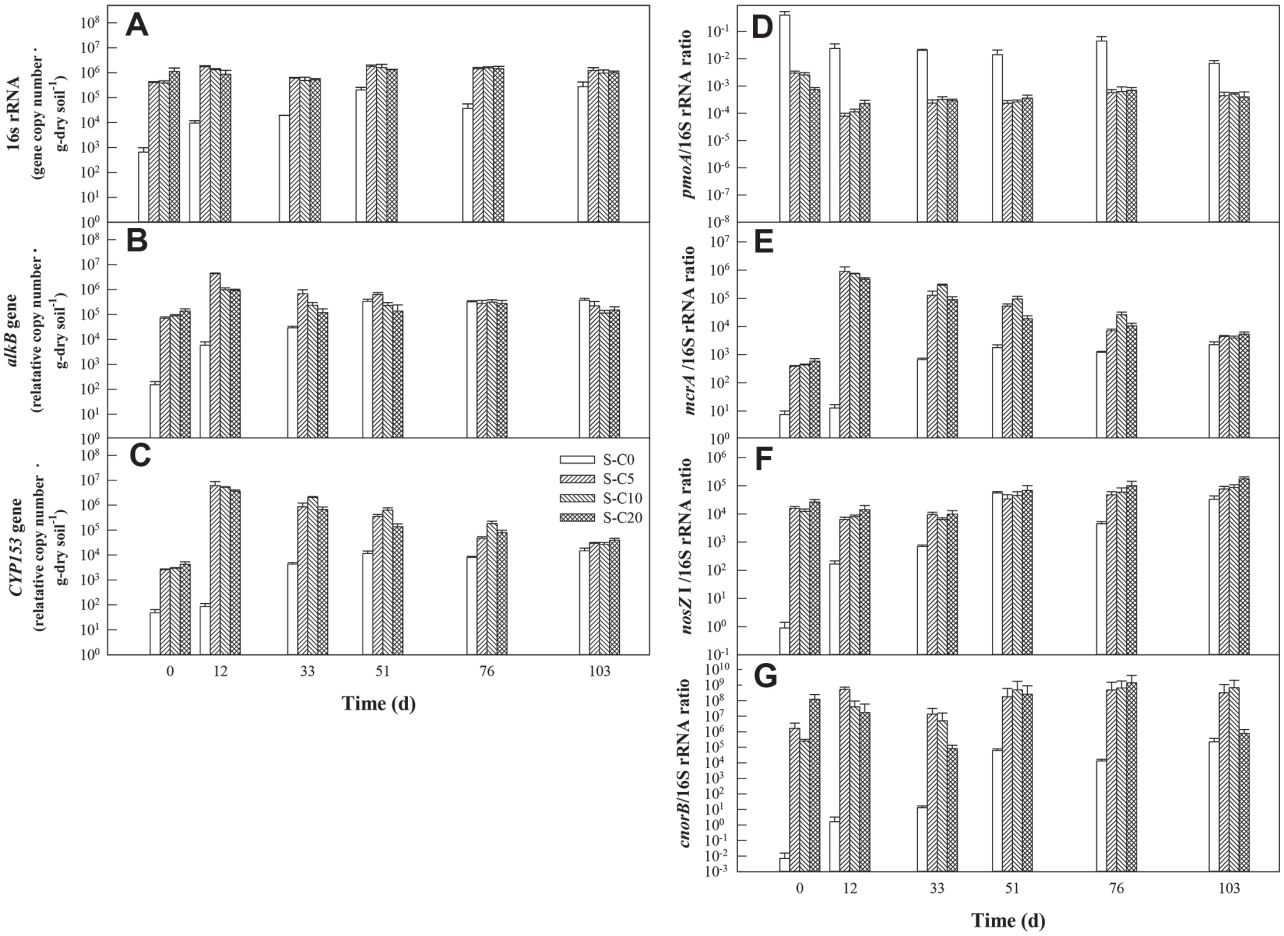
Analysis of functional gene dynamics: (A–C) changes in the copy numbers of (A) 16S rRNA, (B) *alkB*, and (C) *CYP153*; (D–G) changes in the ratios of (D) *pmoA*/16SrRNA, (E) *mcrA*/16SrRNA, (F) *nosZ* I/ 16SrRNA, and (G) *cnorB*/16SrRNA.

The functional gene dynamics during bioremediation of various diesel-contaminated soil samples are indicated in Fig. 3. Thus, the 16S rRNA gene copy number of the S-C0 sample increased from 10^3^ to 10^5^/g dry soil, while those of the S-C5, S-C10, and S-C20 were maintained at around 10^6^/g dry soil during bioremediation (Fig. 3A).

Meanwhile, the relative copy numbers of the *alkB* gene in the S-C0 increased with bioremediation time to match that of the compost-amended soils (gene copy number = 10^5^/g dry soil) at day 51, and remained constant thereafter (Fig. 3B). The relative *alkB* gene copy numbers of the S-C5, S-C10, and S-C20 samples also varied during the initial period (0–33 days), but did not vary significantly after day 51. However, while the relative *CYP153* gene copy number in the S-C0 increased from 10^2^ to 10^4^/g dry soil during bioremediation, those of the S-C5, S-C10, and S-C20 samples decreased from 10^6^ to 10^4^/g dry soil (Fig. 3C). Ultimately, on day 103, the *CYP153* gene copy numbers were similar in all soil samples regardless of compost addition.

The *pmoA*/16S rRNA ratio of the S-C0 was always higher than that of the S-C5, S-C10, and S-C20 during bioremediation (Fig. 3D). The *mcrA*/16S rRNA ratio of the S-C0 sample increased from 10^1^ to 10^3^, whereas that of the S-C5, S-C10, and S-C20 samples increased from 10^2^ to 10^6^ during the initial 12 days, and then gradually decreased to 103 (Fig. 3E). The *nosZ* I/16S rRNA and *cnorB*/16S rRNA ratios in the S-C0 increased until day 51, and remained constant thereafter, while those of the compost-amended soils increased slightly, with some exceptions (Figs. 3F and 3G).

### Bacterial Community Dynamics

**Fig. 4:**
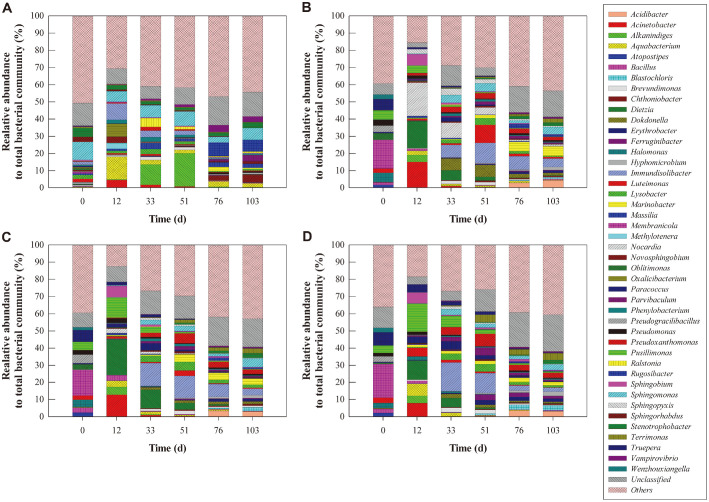
Dynamics of bacterial communities in diesel-contaminated soil at the genus level (A) in the absence of compost (S-C0), and (B–D) during bioremediation with 5% compost (B), 10% compost (C), and 20% compost (D).

The dynamics of the bacterial communities during bioremediation of the diesel-contaminated soil are characterized by the Miseq analysis in Table 4 and Fig. 4. All samples showed good coverages of 0.99 or higher, thereby indicating that the results explain the actual bacterial communities of diesel-contaminated soil effectively (Table 4). The richness and diversity indices of all samples were increased during the bioremediation process, with those of the compost-amended soils being slightly higher than those of the non-compost-amended soil. However, the indices of the compost-amended soil samples were largely identical, regardless of the amount of compost added.

The genus-level analysis in Fig. 4 indicates that the structure of the bacterial community in the compost-amended soil was different from that of the control sample. In the S-C0 sample, *Sphingomonas* remained predominant (10.4–6.7%) throughout the bioremediation, whereas the other major genera changed over time. Thus, *Stenotrophobacter* (5.2%) and *Sphingohabdus* (3.0%) were dominant during the initial period, but were superseded by *Alkanindiges* (11.9%), *Ralstonia* (5.1%), and *Pseudomonas* (3.9%) during the intermediate period, and by *Rugosibacter* (6.8%), *Chthoniobacter* (4.3%), and *Parvibaculum* (3.8%) during the late period. By contrast, the dominant genera in the various compost-amended soil samples were initially *Membranicola* (15.1–19.6%) and *Truepera* (6.5–7.6%), which were superseded by *Immundisolibacter* (7.3–16.9%), *Dietzia* (5.4–10.9%), and *Paracoccus* (2.3–4.8%) during the intermediate period, and by *Sphingomonas* (3.5–5.1%), *Acidibacter* (3.0–4.50%), *Immundisolibacter* (2.2–5.0%), *Marinobacter* (2.0–5.6%) and *Terrimonas* (2.0-4–0%) during the late period.

**Fig. 5:**
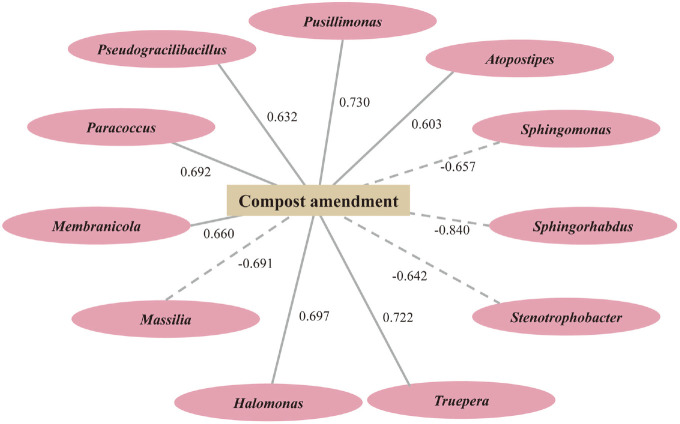
Extended local similarity analysis between compost amendment and bacterial community (*p* < 0.01). The solid line represents a positive relationship between compost amendment and the abundance of the bacterial community. The dashed line represents a negative relationship between compost amendment and the abundance of the bacterial community.

The PCA results in Fig. S5 indicate that the initial bacterial community was clearly divided into two groups according to compost addition, whereas the similarity between the two groups increased as the bioremediation progressed. These results suggest that bacterial community succession in the two groups progressed in a similar direction. Hence, to evaluate the effect of compost amendment upon the structure of the bacterial community, those genera having a close relationship with compost amendment were selected via network analysis (Fig. 5). The results indicate that compost amendment resulted in an increase in the relative abundances of *Atopostipes*, *Halomona*, *Massilia*, *Membranicola*, *Paracoccus*, *Pseudogracilibacillus*, *Pusilimonas*, *Sphingorhabdus*, and *Truepera*, and a decrease in those of *Stenotrophobacter*, *Sphingomonas* and *Massilia*.

## Discussion

### Effects of Compost Amendment on Diesel Removal Efficiency and Soil Enzyme Activity

**Fig. 6:**
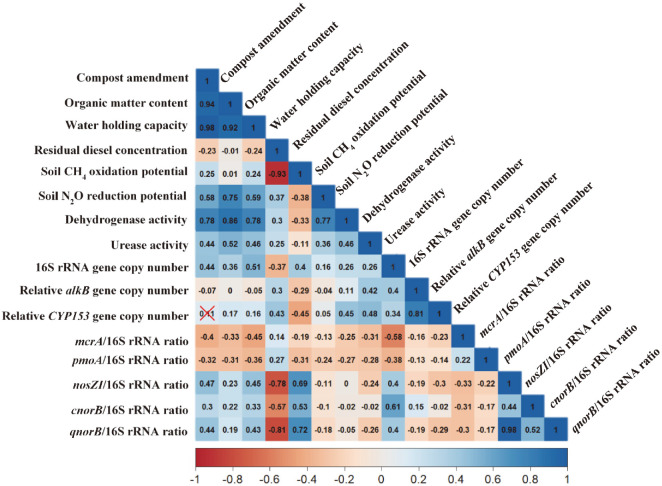
Correlation matrix for all variables. The color bar represents the correlation coefficients from −1 (red) to +1 (blue). The blue squares represent significant positive correlations. The red squares represent significant negative correlations. Darker color tones represent larger correlation coefficients. The red x symbol represents a non-significant correlation coefficient at *p* < 0.05.

The results in Figs. 1 and 6 reveal that the residual diesel concentration is negatively correlated with compost amendment (r = –0.23, *p* < 0.05), thereby indicating a positive correlation between the diesel removal efficiency and compost amendment. However, compost amendment shows an insignificant or weak negative correlation with the functional genes associated with diesel biodegradation (*i.e.*, *alkB* and *CYP153*; Fig. 6). Compost amendment for the bioremediation of diesel-contaminated soil has the effects of supplying nutrients along with a highly diverse microbial community with excellent metabolic potential for diesel degradation [44-46]. Moreover, the compost might have affected the diesel removal efficiency by adsorbing diesel and converting it into an available form for the microorganisms [47, 48]. Humic acids in the compost might act as natural surfactants in the soil, thereby further improving the diesel removal efficiency [49]. However, the compost addition does not always enhance the bioremediation efficiency of oil-contaminated soil. Excessive compost addition can decrease the C/N ratio and thereby inhibit microbial activity [50, 51]. In addition, further study to identify the by-products of diesel during bioremediation is necessary.

The results in Fig. 6 also indicate positive correlations between compost amendment and the DHA (r = 0.78, *p* < 0.05) and UA (r = 0.44, *p* < 0.05) values. Taken together, the results in Figs. 2 and 6 suggest that the DHA and UA are improved because the addition of compost increased the amount of soil organic matter and nutrients, along with the microbial activity. These results are in agreement with previous work by Namkoong *et al*. [52], who noted that the DHA of diesel-contaminated soil increased with the addition of increasing amounts of compost. Other researchers have also reported that the increase in DHA during the bioremediation of oil-contaminated soil is temporary, with a gradual decrease being observed over time [53, 54]. It is well known that the UA has close positive correlations with organic carbon and total nitrogen [55-57].

### Effects of Compost Amendment on the CH_4_ Oxidation and N_2_O Reduction Potentials

All soil samples exhibited CH_4_ oxidation potential regardless of the amount of added compost, and the *pmoA* gene involved in CH_4_ oxidation was also detected in all samples (Table 2, Fig. 3D). Nevertheless, the addition of compost resulted in an enhanced CH_4_ oxidation potential during the bioremediation period (Table 2), and there was a positive correlation between the soil CH_4_ oxidation potential and the compost amendment (Fig. 6). However, the *pmoA*/16S rRNA ratio was higher in the non-compost-amended soil than in the compost-amended soil (Fig. 3D), and the soil CH_4_ oxidation potential showed a negative correlation with the *pmoA* gene (Fig. 6). These results suggest that there is a limit to explaining the CH_4_ oxidation potential only in terms of the behavior of the *pmoA* gene. Bhardwaj and Dubey [58] reported that the concentration of CH_4_-oxidizing bacteria in dry deciduous tropical forest soil had a significant positive correlation with the copy number of the *pmoA* gene (r = 0.9, *p* < 0.01), whereas Qin *et al*. [59] reported no significant relationship between CH_4_-oxidizing bacteria and the *pmoA* gene copy number in acidic paddy soil. In a study by Seo and Cho [18], the compost amendment of diesel-contaminated soil increased the abundance of the *pmoA* gene, but Yang *et al*. [13] noted that the levels of CH_4_ emission were also enhanced by this treatment due to an increased abundance of CH_4_-producing bacteria. Hence, further research is needed to determine the reason for the improvement in the CH_4_ oxidation potential of diesel-contaminated soil by compost addition.

Although the soil CH_4_ production potential was not evaluated in the present study, the dynamics of the CH_4_ production gene, *mcrA*, were monitored. As shown in Fig. 3E, the *mcrA*/16SRNA ratio was increased during bioremediation of the non-compost-amended soil, but decreased in the compost-amended soil. Further, the correlation matrix in Fig. 6 reveals negative correlations between the *mcrA* gene abundance and both the compost amendment and soil CH_4_ oxidation potential. This result suggests that the soil air permeability is improved by the addition of compost, thus making it unfavorable to the growth of anaerobic methanogenic bacteria. This is consistent with previous reports that the addition of exogenous organic matter such as compost can increase the air permeability by increasing the porosity of the soil, thereby improving the removal efficiency of petroleum pollutants [60].

The results in Table 3 and Fig. 6 also reveal positive correlations between compost amendment and both the soil N_2_O reduction potential and the levels of denitrifying genes, such as *nosZI*, *cnorB*, and *qnorB*. In addition, the compost amendment and soil N_2_O reduction potential were each positively correlated with the organic matter content and the DHA. This can be explained by the requirement of carbon and nitrogen sources to act as electron donors and acceptors, respectively, for denitrification metabolism to occur [61]. Moreover, the results in Fig. 6 also reveal strong negative correlations between the residual TPH concentrations and the abundances of *nosZI*, *cnorB*, and *qnorB*. The N_2_O reduction potential of diesel-contaminated soil was higher during the initial stages of bioremediation, and decreased with time as the available carbon and nitrogen sources were consumed (Table 3).

### Bacterial Community Contributing to Diesel Degradation, CH_4_ Oxidation, and N_2_O Reduction

The correlations between the bacterial community, CH_4_ oxidation, and N_2_O reduction are indicated in Table 5. Those genera exhibiting a negative correlation with the residual diesel concentration are associated with diesel degradation. These are *Acidibacter*, *Blastochloris*, *Erythrobacter*, *Hyphomicrobium*, *Marinobacter*, *Parvibaculum*, *Pseudoxanthomonas*, and *Terrimonas*. Interestingly, these bacteria also exhibit a strong positive correlation with the soil CH_4_ oxidation potential. Previous studies have detected *Acidibacter* in soil contaminated with high concentrations (25,000–404,300 mg/kg soil) of petroleum [62]. This genus has also been identified among the dominant bacteria during rhizoremediation of diesel-contaminated soil planted with tall fescue or maize [18]. Meanwhile, *Blastochloris* has been reported as a phototrophic bacterium capable of growing by using aromatic hydrocarbons [63], and has been detected during the rhizoremediation of diesel-contaminated soil [18]. *Erythrobacter* has been shown to degrade petroleum, and its relative abundance was shown to increase with time during the bioaugmentation of petroleum-contaminated seawater [64]. *Hypomicrobium*, a methylotrophic bacterium, was one of the dominant species in a biocover used for the simultaneous removal of CH_4_ and odor [27]. In another study, *Hypomicrobium* was shown to oxidize a high concentration of CH_4_ (100,000 ppm) in a batch reactor [65]. *Marinobacter* has been shown to degrade alkane and polycyclic aromatic hydrocarbons [66, 67] and play a key role in oil degradation during bioremediation [68]. This genus has also been found in frozen soil in the presence of high concentrations of CH_4_ [67]. Meanwhile, Xia *et al*. [69] have reported an increase in the relative abundance of *Parvibaculum* during the remediation of petroleum-contaminated seawater, while Hou *et al*. [70] identified *Pseudoxanthomonas* as one of the dominant rhizobacteria contributing to petroleum degradation during the phytoremediation of contaminated soil using tall fescue. The latter has been shown to degrade diesel in soil [71, 72], and its relative abundance was found to increase during methane oxidation in an anaerobic methane oxidation system [73]. In particular, *Pseudoxanthomonas* sp. Q3 has been isolated as a CH_4_ degrader from a gasfield in China [74]. *Terrimonas* has been identified as one of the active rhizobacteria in the rhizoremediation of diesel-contaminated soil using maize or tall fescue [18]. Meanwhile, Bacosa *et al*. [75] reported that aromatic and aliphatic petroleum compounds were degraded by a bacterial consortium that included *Terrimonas* and Pseudomonax. Taken together, these results of previous studies and those of the present work suggest that *Acidibacter*, *Blastochloris*, *Erythrobacter*, *Hyphomicrobium*, *Marinobacter*, *Parvibaculum*, *Pseudoxanthomonas*, and *Terrimonas* contributed to the diesel degradation and/or CH_4_ oxidation during the bioremediation of the diesel-contaminated soil.

In Table 5, the soil CH_4_ oxidation potential shows a significant positive correlation with *Brevundimonas* and *Ferruginibacter*. Previous studies have described *Brevundimonas* and *Ferruginibacter* as gram-negative heterotrophs [76, 77], but there are no studies on their relevance to CH_4_ oxidation. Hence, future research is needed in order to explain the positive correlation between soil CH_4_ oxidation potential and these bacteria.

In Table 5, the genera exhibiting strong association with soil N_2_O reduction potential are *Atopostipes*, *Bacillus*, *Halomonas*, *Oblitimonas*, *Pusillimonas*, *Truepera*, and *Wenahouziangella*. In particular, *Atopostipes* is negatively correlated with NO_3_ concentration and has been shown to contribute to the denitrification process during the composting of cattle manure [78]. *Bacillus* has been shown to remove nitrate and nitrite by its denitrifying capacity [79, 80]. In particular, inoculation with *Bacillus amyloliquefaciens* has been shown to mitigate N_2_O emission from acidic soil [81]. The aerobic and heterotrophic denitrification capacities of *Halomonas* have been identified and attributed to functional genes such as *napA*, *nirS*, *norB*, and *nosZ* [82]. *Halomonas* has also been shown to contribute to the denitrifying process in an expanded granular sludge bioreactor [83]. Meanwhile, *Pusillimonas* was isolated from nitrate and radionuclide-contaminated groundwater and shown to possess denitrifying functional genes [84]. *Truepera* has been reported as one of the denitrifiers in a sequencing batch biofilm reactor used for landfill leachate treatment [85], and exhibited a high dominance of over 20% in a similar denitrification sequencing batch reactor [86]. The complete denitrification ability of *Wenzhouxiangella* sp. AB-CW3 isolated from a hypersaline soda lake has also been reported [86]. Based on these reports, *Atopostipes*, *Bacillus*, *Halomonas*, *Pusillimonas*, *Truepera*, and *Wenahouziangella* are presumed to have played an important role in the denitrification and/or N_2_O reduction during the bioremediation of diesel-contaminated soil in the present study.

Bioremediation is a promising economical and environmentally friendly soil remediation technology that can be improved by using compost amendment. Herein, diesel-contaminated soil was amended with various weight ratios of compost (0–20%), and correlation and network analyses were used to examine the effects in terms of the dynamics of the bacterial community and functional genes associated with diesel degradation and CH_4_ and N_2_O emission. Thus, compost amendment was positively correlated with the diesel removal efficiency, soil enzyme (dehydrogenase and urease) activity, and soil greenhouse gas (CH_4_ and N_2_O) mitigation capacity via oxidation and reduction, respectively. However, a positive correlation between the compost amendment and functional gene abundance was only detected for the denitrifying genes (*nosZI*, *cnorB*, and *qnorB*) associated with N_2_O reduction. Compost amendment showed weak or insignificant negative correlations with the functional genes associated with diesel biodegradation (*i.e.*, *alkB* and *CYP153*). In addition, compost amendment was negatively correlated with the CH_4_-oxidizing gene *pmoA*. Further detailed studies are needed to determine the reason for the observed mismatch between the activities (diesel degradation and soil CH_4_ oxidation potential) and functional gene abundances (*alkB*, *CYP153*, and *pmoA*).

Network analysis showed that the relative abundances of *Atopostipes*, *Halomona*, *Massilia*, *Membranicola*, *Paracoccus*, *Pseudogracilibacillus*, *Pusilimonas*, *Sphingorhabdus*, and *Truepera* were significantly increased by the compost amendment. Among these genera, *Atopostipes*, *Halomonas*, *Pusillimonas*, and *Truepera* exhibited a strong positive correlation with the soil N_2_O reduction potential. However, the genera that are strongly associated with diesel degradation and soil CH_4_ oxidation potential (*i.e.*, *Acidibacter*, *Blastochloris*, *Erythrobacter*, *Hyphomicrobium*, *Marinobacter*, *Parvibaculum*, *Pseudoxanthomonas* and *Terrimonas*) were not included among those that exhibited increased abundance upon compost amendment. These results suggest that it is necessary to consider the role of bacteria through an integrated interpretation of various data including bacterial abundance.

## Supplemental Materials

Supplementary data for this paper are available on-line only at http://jmb.or.kr.

## Figures and Tables

**Table 1:** Physicochemical properties of the barren soil, the compost, and the various combinations.

	Barren soil	Compost	S-C0	S-C5	S-C10	S-C20
Total nitrogen (%)	BDL*	2.6 ± 0.3	BDL*	0.13	0.26	0.52
NH_4_^+^-N (mg·kg-soil^–1^)	1.8±0.9	465.3 ± 0.2	1.8	24.98	48.15	94.50
NO_3_^–^-N (mg·kg-soil^–1^)	0.7 ± 0.0	34.5 ± 3.8	0.7	2.39	4.08	7.46
Total phosphorus (mg·kg-soil^–1^)	70.8 ± 3.6	11443.8 ± 603.5	70.8	639.45	1208.10	2345.40
pH	8.54 ± 0.14	9.18 ± 0.00	8.52 ± 0.04	8.13 ± 0.01	8.63 ± 0.07	8.87 ±0 .06
Water content (%)	2.89 ± 0.71	55.33 ± 0.52	3.81 ± 0.53	7.11 ± 0.81	7.66 ± 0.87	14.86 ± 0.64
Organic matter (%)	0.24 ± 0.11	36.73 ± 0.25	0.58 ± 0.08	2.80 ± 0.33	4.04 ± 0.51	8.24 ± 0.45
Water holding capacity (%, v/w)	-	-	24.17 ± 2.50	31.67 ± 2.36	38.89 ± 1.57	46.67 ± 1.36
Soil texture	Sand	Sandy loam	–	–	–	–
Sand (%)	96.68	75.8	–	–	–	–
Silt (%)	1.32	6.44	–	–	–	–
Clay (%)	2	17.76	–	–	–	–

*Below detection limit

**Table 2:** Comparison of soil CH_4_ oxidation potentials.

Time (d)	Soil CH_4_ oxidation potential (μmol·g-dry-soil^–1^·h^–1^)			

	S-C0	S-C5	S-C10	S-C20
0	1.40 ± 0.14 ^I^	1.80 ± 0.04 ^I^	1.72 ± 0.21 ^I^	1.95 ± 0.29 ^I^
12	3.31 ± 0.17 ^H^	1.94 ± 0.50 ^I^	1.60 ± 0.02 ^I^	2.04 ± 0.01 ^I^
33	5.09 ± 0.25 ^F,^ ^G^	4.50 ± 0.61 ^G^	5.02 ± 0.27 ^F,^ ^G^	7.12 ± 0.69 ^D,^ ^E^
51	5.80 ± 0.62 ^F^	7.85 ± 0.20 ^C,^ ^D,^ ^E^	7.00 ± 1.37 ^E^	8.88 ± 0.31 ^B^
76	5.50 ± 0.35 ^F,^ ^G^	8.00 ± 0.11 ^I^	8.21 ± 0.08 ^B,^ ^C^	9.83 ± 0.15 ^A^
103	5.39 ± 0.18 ^F,^ ^G^	7.90 ± 0.06 ^C,^ ^D^	7.78 ± 0.22 ^D,^ ^C,^ ^E^	8.49 ± 0.17 ^B,^ ^C^

**Table 3:** Comparison of the soil N_2_O reduction potentials.

Time (d)	Soil N_2_O reduction potential (nmol·g-dry-soil^–1^·h^–1^)			

	S-C0	S-C5	S-C10	S-C20
0	< 56.8 ± 4.8	868.0 ± 44.4^C^	1,399.6 ± 18.5^B^	1,757.8± 7.3^A^
12	< 56.8 ± 4.8	329.6 ± 54.7^F,^ ^G,^ ^H^	372.1 ± 19.7^F^	838.1 ± 36.9^C^
33	< 56.8 ± 4.8	316.2 ± 19.5^I,^ ^G,^ ^H^	281.7 ± 38.7^I,^ ^J,^ ^H^	603.6 ± 4.1^E^
51	< 56.8 ± 4.8	265.4 ± 17.4^I,^ ^J,^ ^K^	363.4 ± 20.1^F,^ ^G^	661.8 ± 50.3^D^
76	< 56.8 ± 4.8	250.3 ± 4.8^J,^ ^K^	256.0 ± 14.9^J,^ ^K^	275.3 ± 31.0^I,^ ^J,^ ^H^
103	< 56.8 ± 4.8	140.4 ± 3.4^J,^ ^K^	214.9 ± 9.1^K^	224.1 ± 5.7^J,^ ^K^

**Table 4:** The richness and diversity of the bacterial community.

Soil sample	Time (d)	OTU	Chao1^a^	Shannon^b^	Inverse Simpson^c^	Good's Coverage^d^
S-C0	0	297 ± 1	1249.4 ± 0.8	7.67 ± 0.03	0.989 ± 0.000	0.997 ± 0.000
	12	201 ± 10	954.9 ± 1.3	6.29 ± 0.02	0.965 ± 0.002	0.996 ± 0.001
	33	251 ± 1	1180.7 ± 1.6	6.69 ± 0.00	0.974 ± 0.000	0.997 ± 0.000
	51	322 ± 40	1484.3 ± 148.9	6.81 ± 0.05	0.955 ± 0.003	0.995 ± 0.002
	76	554 ±32	2534.4 ± 114.0	8.03 ± 0.02	0.987 ± 0.001	0.996 ± 0.001
	103	485 ± 45	2097.0 ± 146.1	8.03 ± 0.04	0.988 ± 0.000	0.994 ± 0.002
S-C5	0	176 ± 11	828.1 ± 7.5	6.35 ± 0.01	0.964 ± 0.001	0.998 ± 0.001
	12	149 ±18	691.5 ± 85.4	4.89 ± 0.13	0.913 ± 0.009	0.998 ± 0.001
	33	205 ± 9	927.1 ± 47.1	6.56 ± 0.01	0.974 ± 0.001	0.998 ± 0.000
	51	265 ±6	1216.4 ± 3.7	6.39 ± 0.01	0.963 ± 0.000	0.997 ± 0.000
	76	553 ±34	2361.9 ± 102.6	8.06 ± 0.03	0.987 ± 0.000	0.995 ± 0.001
	103	638 ±53	2626.5 ± 203.0	8.42 ± 0.01	0.990 ± 0.000	0.995 ± 0.002
S-C10	0	172 ± 9	786.0 ± 45.8	6.48 ± 0.05	0.969 ± 0.002	0.998 ± 0.000
	12	156 ±7	689.1 ± 43.8	5.20 ± 0.05	0.930 ± 0.004	0.999 ± 0.000
	33	219 ±17	975.1 ± 28.7	6.41 ± 0.04	0.962 ± 0.002	0.998 ± 0.001
	51	341 ±6	1440.1 ± 24.6	6.89 ± 0.01	0.972 ± 0.000	0.999 ± 0.000
	76	634 ± 48	2581.2 ± 87.8	8.24 ± 0.02	0.989 ± 0.000	0.996 ± 0.001
	103	702 ± 9	2826.0 ± 31.0	8.57 ± 0.01	0.992 ± 0.000	0.996 ± 0.000
S-C20	0	174 ± 18	702.7 ± 57.4	6.30 ± 0.13	0.956 ± 0.007	0.998 ± 0.001
	12	163 ± 12	627.8 ± 27.3	5.50 ± 0.03	0.952 ± 0.001	0.999 ± 0.000
	33	217 ± 6	916.0 ± 48.8	6.24 ± 0.03	0.958 ± 0.001	0.998 ± 0.000
	51	325 ± 17	1234.1 ± 78.1	6.83 ± 0.03	0.974 ± 0.001	0.997 ± 0.000
	76	717 ± 18	2495.9 ± 31.5	8.38 ± 0.00	0.992 ± 0.000	0.995 ± 0.001
	103	667 ± 14	2506.4 ± 38.7	8.58 ± 0.02	0.993 ± 0.000	0.994 ± 0.000

^a^The Chao1 index is used to evaluate the bacterial population richness.

^b^The Shannon index is used to evaluate the diversity within the bacterial population. It accounts for both species abundance and evenness.

^c^The Simpson diversity index is calculated as D = 1–[Σn(n–1)/N(N–1)], where n is the number of individuals of each species and N is the total number of individuals of all species. The Simpson diversity index is the probability that two randomly selected individuals in a given habitat will belong to the same species.

^d^Good coverage is calculated as C = 1– (s/n), where s is the number of unique operational taxonomic units (OTUs) and n is the number of individuals of each species. The index gives a relative measure of how well the sample represents a large environment.

**Table 5:** Correlation matrix of the bacterial community, residual diesel concentration, soil CH_4_ oxidation potential, and soil N_2_O reduction potential (*p* < 0.05).

Genus	Residual diesel^a^	CH_4_ oxidation^b^	N_2_O reduction^c^	Genus	Residual diesel^a^	CH_4_ oxidation^b^	N_2_O reduction^c^
*Acidibacter*	**–0.780**	**0.663**	–0.223	*Nocardia*	0.322	–0.252	–
*Acinetobacter*	0.504	–0.505	–0.019	*Novosphingobium*	0.214	–0.086	–0.389
*Alkanindiges*	0.148	–0.129	–0.238	*Oblitimonas*	0.494	–0.54	**0.559**
*Aquabacterium*	0.392	–0.324	–0.228	*Oxalicibacterium*	0.291	–0.195	–0.205
*Atopostipes*	0.460	–0.517	**0.860**	*Paracoccus*	0.197	–0.075	0.242
*Bacillus*	0.562	–0.668	**0.712**	*Parvibaculum*	**–0.444**	**0.543**	–0.107
*Blastochloris*	**–0.748**	**0.643**	–0.159	*Phenylobacterium*	0.000	0.137	–0.528
*Brevundimonas*	–0.151	**0.428**	–0.202	*Pseudogracilibacillus*	0.436	–0.493	**0.806**
*Chthoniobacter*	–0.400	0.288	–0.369	*Pseudomonas*	0.534	–0.468	–0.031
*Dietzia*	0.404	–0.405	0.043	*Pseudoxanthomonas*	**–0.540**	**0.73**	–0.119
*Dokdonella*	–0.149	0.336	–0.050	*Pusillimonas*	0.495	–0.518	**0.508**
*Erythrobacter*	**–0.794**	**0.802**	–0.123	*Ralstonia*	-0.009	0.044	–0.308
*Ferruginibacter*	**–0.564**	**0.612**	–0.022	*Rugosibacter*	-0.307	0.18	–0.328
*Halomonas*	0.511	–0.553	**0.800**	*Sphingobium*	0.379	–0.411	0.053
*Hyphomicrobium*	**–0.746**	**0.594**	–0.350	*Sphingomonas*	–0.097	0.127	**–0.668**
*Immundisolibacter*	–0.370	**0.568**	–0.051	*Sphingopyxis*	0.094	0.091	–0.089
*Luteimonas*	0.206	–0.090	0.279	*Sphingorhabdus*	0.313	–0.329	–0.324
*Lysobacter*	–0.283	**0.490**	–0.268	*Stenotrophobacter*	-0.235	0.126	–0.611
*Marinobacter*	**–0.753**	**0.715**	–0.236	*Terrimonas*	**–0.773**	**0.768**	–0.165
*Massilia*	0.129	–0.150	–0.512	*Truepera*	0.519	–0.554	**0.946**
*Membranicola*	0.433	–0.496	**0.836**	*Vampirovibrio*	–0.314	0.206	–0.457
*Methylotenera*	0.247	–0.148	–0.209	*Wenzhouxiangella*	0.405	–0.475	**0.786**

^a^Correlation coefficients between residual diesel concentration and bacterial abundance.

^b^Correlation coefficients between CH_4_ oxidation potential rate and bacterial abundance.

^c^Correlation coefficients between N_2_O reduction potential rate and bacterial abundance.
