# Supplementary material for: Dynamics of Functional Genes and Bacterial Community during Bioremediation of Diesel-Contaminated Soil Amended with Compost
Source: J Microbiol Biotechnol. 2023 Feb 10;33(4):471–84. doi: 10.4014/jmb.2210.10038 (PMC10164733; doi:10.4014/jmb.2210.10038)
Supplement: Supplementary file 1 [file jmb-33-4-471-supple.pdf]

J. Microbiol. Biotechnol.
https://doi.org/10.4014/jmb.2210.10038

Supplementary Figures and Table

Table S1.

The primer sets and qPCR conditions for the analysis of functional genes.

Functional gene	Coding enzyme	Primer	Sequence (5'-3')	qPCR conditions	Reference
16S rRNA	16S rRNA sequence of *E. coli*	340F<br>805R	TCC TAC GGG AGG CAG CAG<br>GAC TAC HVG GGT ATC TAA TCC	95℃ 3 min;<br>95℃ 15 s, 50 ℃ 30 s, 72 ℃ 30 s; 40 cyc<br>82 ℃ 30 s	Kim et al., 2012
alkB	Rubredoxin dependent alkane monooxygenase of *Pseudomoans. putida* GPo1	alkB-1F<br>alkB-1R	AAYACNGCNCAYGARCTNGGNCAYAA<br>GCRTGRTGRTCNGARTGNCGYTG	95 ℃ 2 min;<br>95 ℃ 20 s, 55 ℃ 20 s, 72 ℃ 40 s; 40 cyc<br>82 ℃ 30 s	Wasmund et al., 2009
CYP153	Cytochrome P450 alkane hydroxylase of *Acinetobacter* sp. EB104	CYP153_C4_F<br>CYP153_C4_R	CGA GAA GCT CAA GTC CAA CC<br>CAC ATC ACG ACC TTG TCA CC	95 ℃ 5 min;<br>95 ℃ 30 s, 58 ℃ 30 s, 72 ℃ 30 s; 40 cyc<br>82 ℃ 30 s	Alonso-Gutierrez et al., 2011
pmoA	Alpha subunit of particulate methane monooxygenase of *Methylobacter* and *Methylomicrobium*	A189f<br>mb661r	GGN GAC TGG GAC TTC TGG<br>CCG GMG CAA CGT CYT TAC C	95 ℃ 3 min;<br>95 ℃ 15 s, 63 ℃ 30 s, 72 ℃ 30 s; 40 cyc<br>82 ℃ 30 s	Kolb et al., 2003
mcrA	Alpha subunit of methyl coenzyme-M reductase of Methanogenic archaea	mlas<br>mcrA-rev	GGT GGT GTM GGD TTC ACM CAR TA<br>CGT TCA TBG CGT AGT TVG GRT AGT	95 ℃ 3 min;<br>95 ℃ 30 s, 50 ℃ 40 s, 72 ℃ 40 s; 35 cyc<br>82 ℃ 30s	Luton et al., 2002
qnorB	Subunit B of nitric oxide reductase of *Ralstonia eutropha* H16	qnorB-2F<br>qnorB-7R	GGN CAY CAR GGN TAY GA<br>GGN GGR TTD ATC ADG AAN CC	95 ℃ 5 min;<br>95 ℃ 30 s, 51 ℃ 30 s, 72 ℃ 60 s; 40 cyc<br>82 ℃ 30 s	Braker et al., 2003
cnorB	Subunit B of nitric oxide reductase of *Paracoccus denitrificans* Pd1222	norB1-F<br>norB6-R	CGN GAR TTY CTS GAR CAR CC<br>TGC AKS ARR CCC CAB ACB CC	95 ℃ 5 min;<br>95 ℃ 30 s, 50 ℃ 30 s, 72 ℃ 60 s; 40 cyc<br>82 ℃ 30 s	Casciotti et al., 2005
nosZ Ⅰ	Subunit Z of nitrous oxide reductase of *Wolinella*	nosZ 1F<br>nosZ 1R	WCS YTG TTC MTC GAC AGC CAG<br>ATG TCG ATC ARC TGV KCR TTY TC	95 ℃ 5 min;<br>95 ℃ 30 s, 56 ℃ 30 s, 72 ℃ 60 s; 40 cyc<br>82 ℃ 30 s	Henry et al., 2006

succinogenes

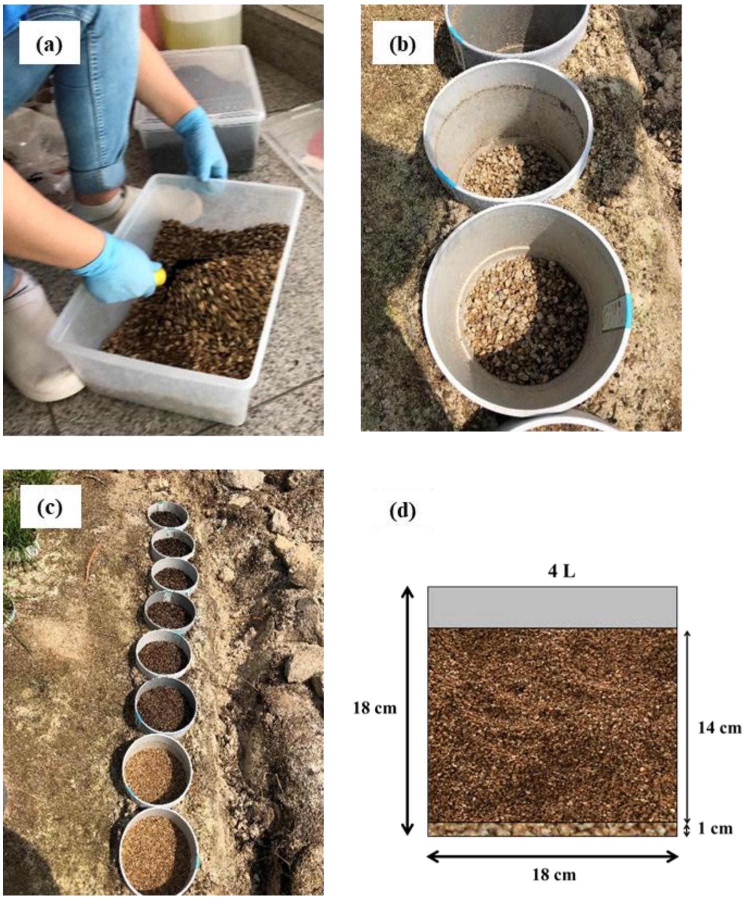

Fig. S1. **Soil preparation and pot setting**: (a) mixing the soil, compost, and diesel; (b) the layers of coarse sand in the pots; (c) the pots partially buried in the garden soil; (d) a schematic cross section of a pot containing the sand and soil.

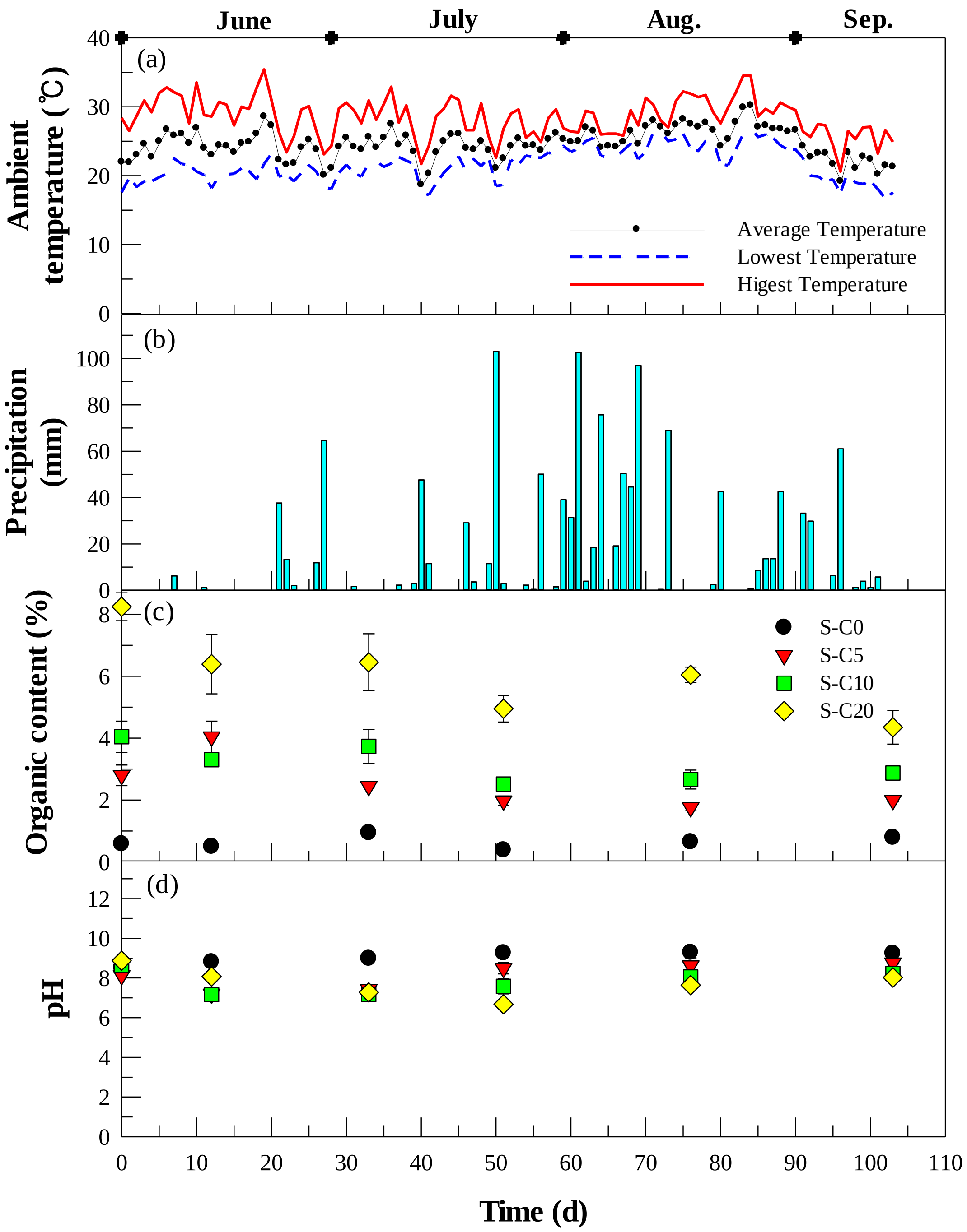

Fig. S2. **The changes in (a) ambient temperature, (b) precipitation, (c) soil organic content, and (d) soil pH with time during the bioremediation of the diesel-contaminated soils.** S-C0 = soil without compost; S-C5 = soil with 5% compost; S-C10 = soil with 10% compost; S-C20 = soil with 20% compost.

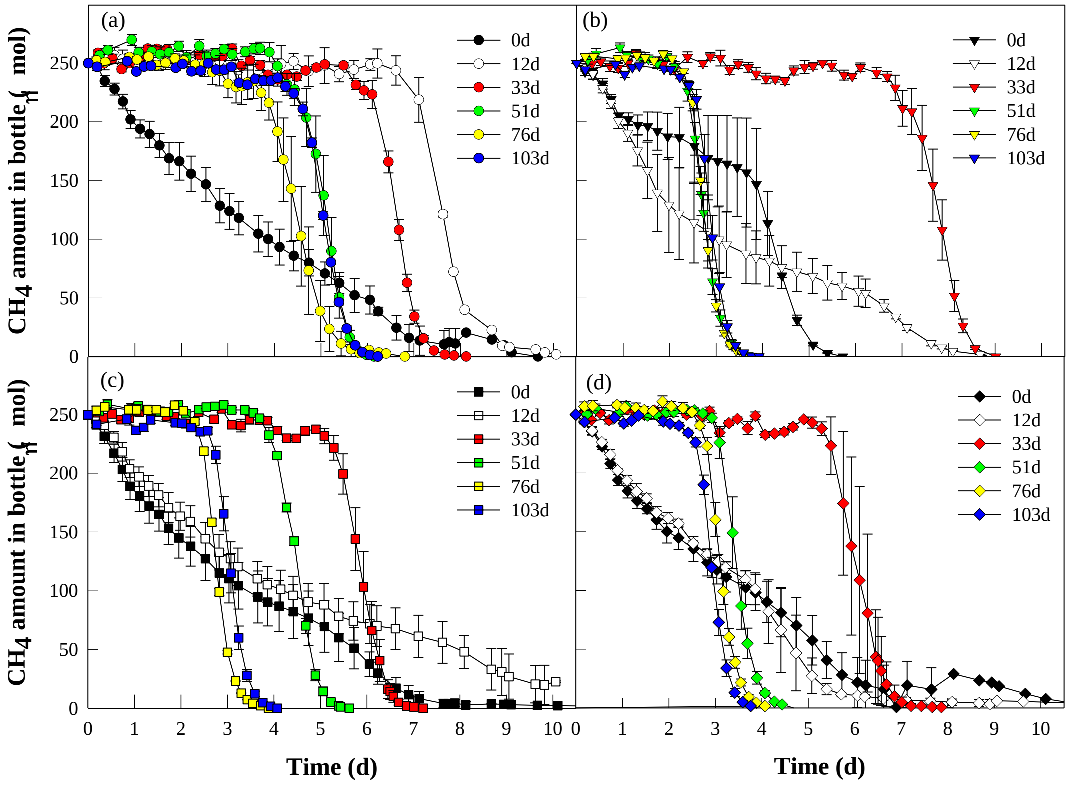

Fig. S3. **The changes in CH_4 concentration in the headspace of the serum bottle inoculated with the various soil samples**: (a) S-C0 (soil without compost); (b) S-C5 (soil with 5% compost); (c) S-C10 (soil with 10% compost); (d) S-C20 (soil with 20% compost).

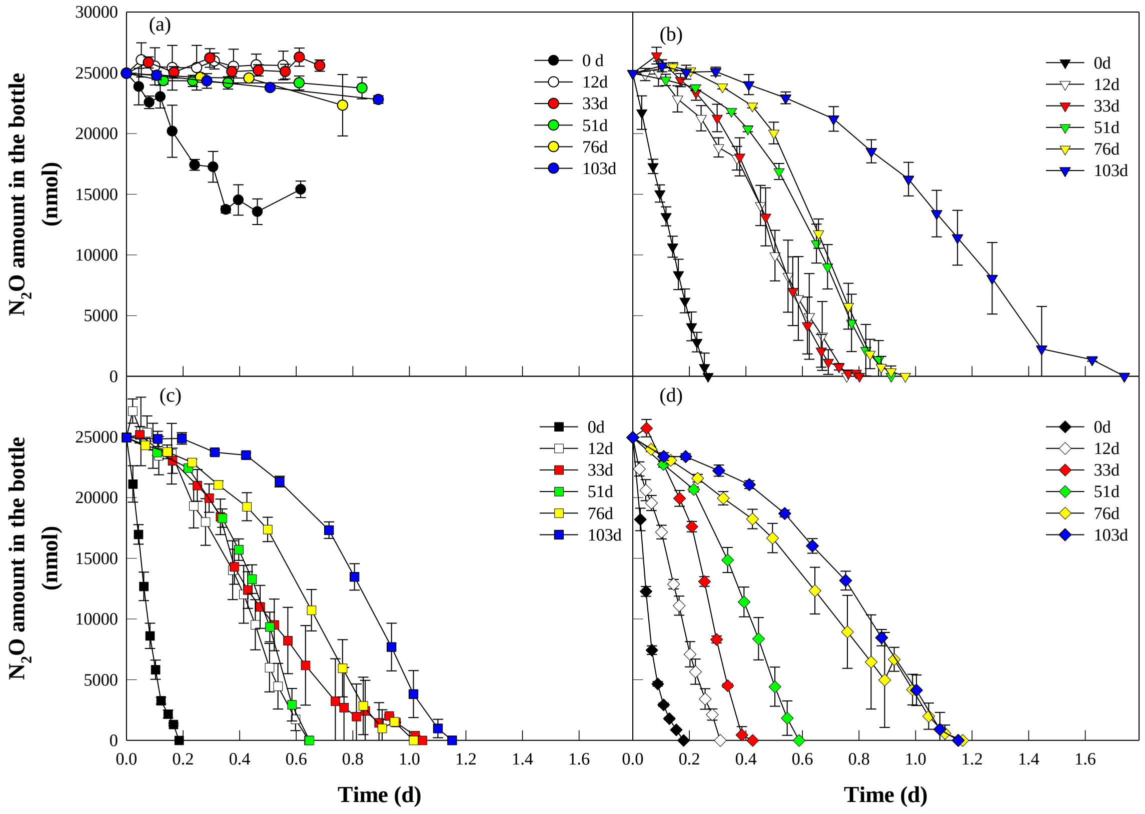

Fig. S4. **The changes in the N_2O concentration in the headspace of the serum bottle inoculated with soil samples**: (a) S-C0 (soil without compost); (b) S-C5 (soil with 5% compost); (c) S-C10 (soil with 10% compost); (d) S-C20 (soil with 20% compost).

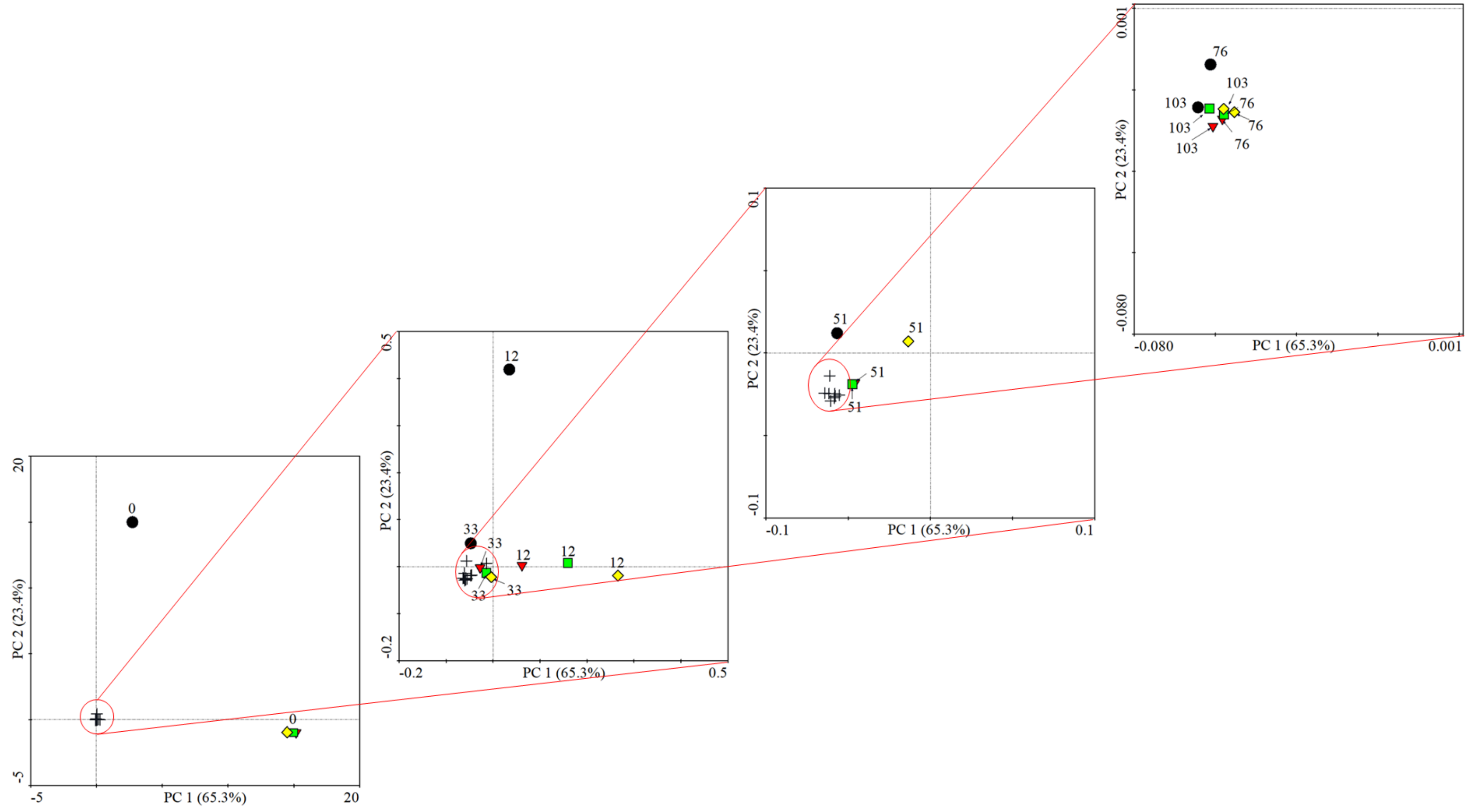

Fig. S5. **Principal component analysis (PCA) of the bacterial community structure at the genus level in the diesel-contaminated soil.** The community structure was analyzed in duplicate. The circles represent the soil without compost (S-C0), the inverted triangles represent the soil with 5% compost (S-C5), the squares represent the soil with 10% compost (S-C10), and the diamonds represent the soil with 20% compost (S-C20).
